# Sodium selectivity of semicircular canal duct epithelial cells

**DOI:** 10.1186/1756-0500-4-355

**Published:** 2011-09-13

**Authors:** Muneharu Yamazaki, Tao Wu, Satyanarayana R Pondugula, Donald G Harbidge, Daniel C Marcus

**Affiliations:** 1 Cellular Biophysics Laboratory, Department of Anatomy & Physiology, Kansas State University, Manhattan, KS 66506, USA; 2 Department of Otolaryngology-Head and Neck Surgery, Tohoku University Graduate School of Medicine, Sendai, Japan 980-8574

## Abstract

**Background:**

Sodium absorption by semicircular canal duct (SCCD) epithelial cells is thought to contribute to the homeostasis of the volume of vestibular endolymph. It was previously shown that the epithelial cells could absorb Na^+ ^under control of a glucocorticoid hormone (dexamethasone) and the absorptive transepithelial current was blocked by amiloride. The most commonly-observed target of amiloride is the epithelial sodium channel (ENaC), comprised of the three subunits α-, β- and γ-ENaC. However, other cation channels have also been observed to be sensitive in a similar concentration range. The aim of this study was to determine whether SCCD epithelial cells absorb only Na^+ ^or also K^+ ^through an amiloride-sensitive pathway. Parasensory K^+ ^absorption could contribute to regulation of the transduction current through hair cells, as found to occur via vestibular transitional cells [S. H. Kim and D. C. Marcus. Regulation of sodium transport in the inner ear. *Hear.Res*. doi:10.1016/j.heares.2011.05.003, 2011].

**Results:**

We determined the molecular and functional expression of candidate cation channels with gene array (GEO GSE6197), whole-cell patch clamp and transepithelial recordings in primary cultures of rat SCCD. α-, β- and γ-ENaC were all previously reported as present. The selectivity of the amiloride-sensitive transepithelial and cell membrane currents was observed in Ussing chamber and whole-cell patch clamp recordings. The cell membrane currents were carried by Na^+ ^but not K^+^, but the Na^+ ^selectivity disappeared when the cells were cultured on impermeable supports. Transepithelial currents across SCCD were also carried exclusively by Na^+^.

**Conclusions:**

These results are consistent with the amiloride-sensitive absorptive flux of SCCD mediated by a highly Na^+^-selective channel, likely αβγ-ENaC. These epithelial cells therefore absorb only Na^+ ^via the amiloride-sensitive pathway and do not provide a parasensory K^+ ^efflux from the canals via this pathway. The results further provide caution to the culture of epithelial cells on impermeable surfaces.

## Background

Hearing and balance depend on the ion homeostasis of the luminal fluid, endolymph [1,2]. Transduction of stimulus into neuronal signals is mediated and regulated predominantly by K^+ ^and Ca^2+^; however, maintenance of a very low [Na^+^] is also critical to prevent a toxic overload of the sensory hair cells (e.g.,[3]) and for osmotic balance. Cellular Na^+ ^absorptive mechanisms have been observed in the cochlea and vestibular labyrinth [4]. The primary Na^+ ^transport pathways are mediated by an amiloride- and benzamil-sensitive ion channel and by a nonselective cation channel. The former is found in Reissner's membrane, saccule and semicircular canal duct (SCCD). The most commonly-observed target of these drugs is the epithelial sodium channel (ENaC), comprised of the three subunits α-, β- and γ-ENaC.

The cation selectivity of the amiloride- and benzamil-sensitive ion channel is of interest since 1) nonselective and poorly-selective cation channels have also been observed to be sensitive in a similar concentration range of amiloride and benzamil (see Discussion) and 2) the high concentration of endolymphatic K^+ ^would lead to an important efflux of K^+ ^through these cells. The high Na^+ ^selectivity of epithelial cells in Reissner's membrane was recently reported [5]; in the present study we also found a high sodium selectivity of epithelial cells of the SCCD. The potential influence of the permeability of the culture support on ion channel expression was also investigated and found to have a profound effect.

## Results

### SCCD epithelial cells grown on permeable supports

#### Transepithelial absorption of Na^+ ^and K^+ ^(Ussing chamber recordings)

**Figure 1:**
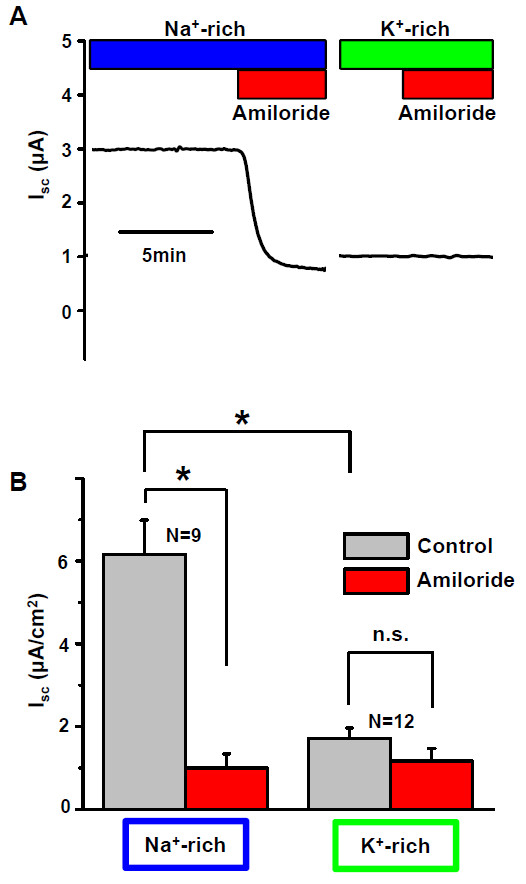
**Short circuit current (I_sc_) across primary cultures of semicircular canal duct (SCCD) epithelial cells grown on permeable supports**. A) Representative traces. Basolateral solution was physiological saline and apical solution was either Na^+^-rich (*left*) or K^+^-rich (*right*). Note break in time axis; Isc of each culture insert was measured in each apical solution using different order of exposure and washing in between measurements. Amiloride (100 μM) added on the apical side as indicated by horizontal red bar. B) Summary of normalized currents from experiments as in A.

Transepithelial cation absorption was measured as the short-circuit current (I_sc_) across the epithelium from the apical side towards the basolateral side under conditions where the only major permeant ions on the apical side were either Na^+ ^or K^+ ^(Figure 1). In Na^+^-rich solution on the apical side, amiloride (100 μM) significantly inhibited the I_sc _by 84% (Table 1) when the cells were grown on permeable supports. I_sc _in K^+^-rich solution in the absence of amiloride was significantly smaller than in Na^+^-rich solution (Figure 1B). In K^+^-rich solution on the apical side, amiloride had no significant effect on the I_sc _(Figure 1B, Table 1). A representative experiment is shown in Figure 1A and a summary of similar experiments is shown in Figure 1B. In K^+^-rich solution, I_sc _was under 1 μA/cm^2 ^in 11 experiments, but in one experiment was 3.16 μA/cm^2^. The single sample with the large current was likely due to a culture support with fewer perforations (lower permeability) than usual.

**Table 1:** SCCD transepithelial and cellular currents

		Na^+^-rich		K^+^-rich	
			
		control	amiloride	control	amiloride
permeable	Ussing chamber (I_sc_, μA/cm^2^)	6.16 ± 0.83 (9)	1.00 ± 0.34 (9)*	1.71 ± 0.26 (12)	1.17 ± 0.3 (12) ns
			
	Whole cell patch (I_-120 mV_, pA/pF)	-60.8 ± 20.3 (9)	-36.3 ± 17.3 (9)*	-3.8 ± 0.1 (5)	-3.6 ± 0.3 (5) ns

impermeable (I_-120 mV_, pA/pF)	Whole cell patch	-137.6 ± 68.7 (6)	-127.9 ± 64.2 (6) ns	-69.6 ± 6.1 (7)	-64.0 ± 5.7 (7)*

Table entries are means ± SEM (number of experiments).

I_sc _: short circuit current (μA/cm^2^), I_-120 mV _: whole-cell patch clamp current at -120 mV command voltage (pA/pF)

Amilorde concentration was 100 μM for all experiments.

Significant differences: *, P < 0.05; ns, not significant.

Comparisons: Values in amiloride *vs *control (without amiloride) using the paired t-test.

#### Cellular cation currents (whole-cell patch clamp recordings)

**Figure 2:**
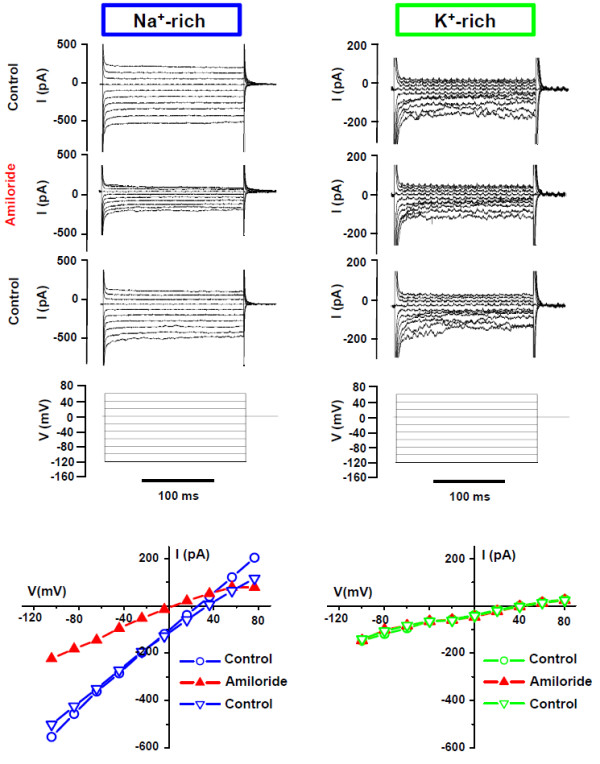
**Representative whole-cell patch clamp recordings and current-voltage curves from SCCD epithelial cells grown on permeable supports**. *Left panel*, Na^+ ^-rich bath and pipette, *Right panel*, K^+^-rich bath and pipette. The holding potential and voltage command protocol are illustrated. Amiloride concentration was 100 μM. *Bottom*: Representative current-voltage relationships of the whole cell currents in the presence and absence of amiloride (100 μM). Voltages are corrected for the liquid junction potentials (see Methods).

**Figure 3:**
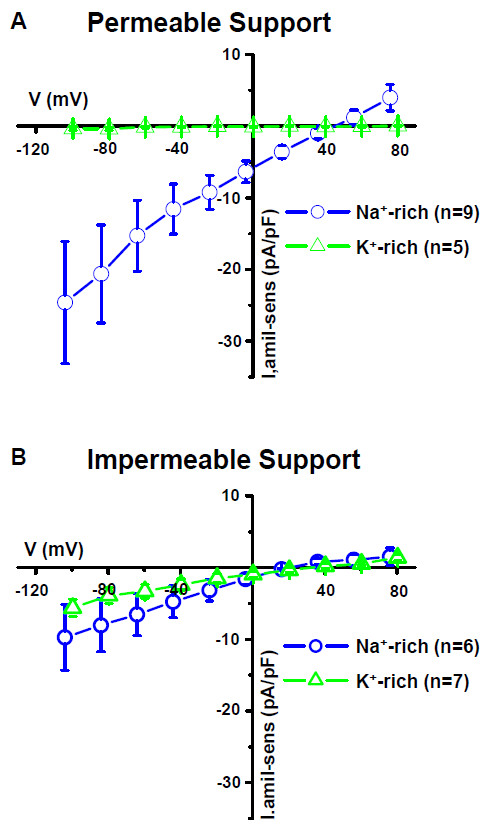
**Summary of current-voltage relationships of amiloride-sensitive currents on permeable and impermeable supports**. Summary of current-voltage relationships in terms of amiloride-sensitive currents measured in Na^+^-rich bath solution and K^+^-rich bath solution from SCCD epithelial cells grown on (A) permeable supports and (B) impermeable supports. Voltages are corrected for the liquid junction potentials (see Methods). I, amil-sens, amiloride-sensitive currents (pA/pF).

**Figure 4:**
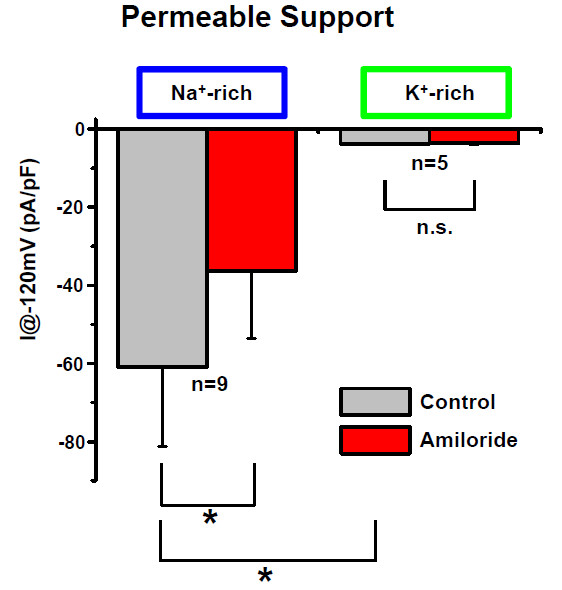
**Summary of whole-cell patch clamp currents with only Na^+ ^and K^+ ^as the major permeant ions**. Bar graph of the currents at -120 mV in Na^+ ^-rich solution and K^+^-rich solutions. Amiloride-sensitive currents were only observed in Na^+ ^-rich solution.

The currents were measured under conditions where the only major permeant ions were either Na^+ ^or K^+^; Cl^- ^was replaced by gluconate. (Figures 2, 3A, 4). In Na^+^-rich bath solution, amiloride (100 μM) significantly inhibited the inward whole-cell current (carried mostly by bath Na^+ ^at -120 mV) by 59.6% (Table 1). A representative experiment is shown in Figure 2 and 2a summary of similar experiments is shown in Figure 3A and 4. The reversal voltage in the absence of amiloride reflects the asymmetrical [Na^+^] and the leftward shift of the reversal voltage in the presence of amiloride is consistent with the block of a Na^+^-permeable channel. These whole-cell data are consistent with the observations of Na^+^-selective I_sc _(above). In K^+^-rich bath solutions, the current at -120 mV was significantly smaller than in Na^+ ^(Figure 4). Amiloride (100 μM) had no significant effect on the reversal voltage nor on the inward whole-cell current (carried mostly by bath K^+ ^at -120 mV) (Table 1). A representative experiment is shown in Figure 2 and a summary of similar experiments is shown in Figure 3A and 4.

### SCCD epithelial cells grown on impermeable supports

#### Cellular cation currents (whole-cell patch clamp recordings)

When primary SCCD cells were grown on impermeable supports (glass coverslips) and were bathed in Na^+^-rich solution, amiloride (100 μM) had no significant effect on the inward whole-cell current (carried mostly by bath Na^+ ^at -120 mV; Figure 3B, Table 1). In K^+^-rich bath solution, amiloride (100 μM) led to a small inhibition of the inward whole-cell current (carried mostly by bath K^+ ^at -120 mV) by 8.1% (Table 1). There was no significant difference in the amiloride-sensitive inward current between the Na^+^- rich bath at -120 mV (I_-120 _= -9.7 ± 4.6 pA/pF, n = 6) and K^+^-rich bath solutions (I_-120 _= -5.6 ± 1.2 pA/pF, n = 7) (Figure 3B). It was observed that culture on impermeable supports led to no significant change in Na^+ ^-current, but to a marked increase in the K-current (factor of 18) (Figure 3, Table 1). These results are consistent with the expression of amiloride-insensitive, nonselective cation channels (see Discussion).

### Expression of amiloride-sensitive cation channels in SCCD

We utilized gene array (GEO GSE6197; Table 2) [6,7] to partially address the question of the participation of amiloride-sensitive nonselective cation channels. Several isoforms of acid-sensitive ion channels (ASIC) and cyclic-nucleotide gated (CNG) channels were listed in our gene array of rat SCCD. We examined whether each gene was present or absent in primary cultures of SCCD after incubation for 24 h with 100 nM dexamethasone. ASIC1a, 2a were listed; ASIC1a had a call of "Present", but ASIC2a was "Absent" (Table 2). CNGA1, 2, 3 were listed in the gene array and CNGA1 yielded a call of "Present", but CNGA2, 3 each received a call of "Absent". HCN1, 2, 3, 4 were listed; HCN1, 3 had a call of "Present", but HCN 2, 4 were both "Absent" (Table 2). ENaC can be an nonselective cation channel under some subunit combinations (see Discussion) and the α-, β- and γ-ENaC subunits were all expressed in SCCD cultured on permeable supports, as determined by gene array, RT-PCR and sequence validation [6-8] (Table 2).

**Table 2:** Amiloride sensitive channels

name	gene bank	gene	amiloride, IC_50_, μM	cation selectivity, P_Na_/P_K_	gene array*, DEX (GEO, GSE6197)	reference
α-ENaC	NM_031548	Scnn1a	0.17	1	P	[15,14,20,21]
αβ-ENaC	U35174	Scnn1b	4	≥ 100	P	[18,19]
αγ-ENaC	U37539	Scnn1g	0.13	≥ 100	P	[18,19]
αβγ-ENaC			0.1	≥ 100	P	[16,18,14]
ASIC2a	Y14635	Accn1	28	≥ 10	A	[18,31]
ASIC1a	NM_024154	Accn2	10	6-13	P	[18,31,32]
CNGA1	NM_012923	Ccng1	(100 tested)**	1	P	[23-25,27]
CNGA2	AF_126808	Cncg4	ND	1	A	[24,27,26]
CNGA3	AJ272429	Cnga3	ND	1	A	[24,27,26]
HCN1	NM_053375	Hcn1	ND	0.3	P	[28,29,35]
HCN2	AI112487, AW532988	Hcn2	ND	0.3	A	[28,29,35]
HCN3	NM_053685	Hcn3	ND	0.3	P	[28,29,35]
HCN4	AF247453	Hcn4	ND	0.3	A	[28,29,35]
TRPV1	AB015231, AF158248, AB041029	Trpv1	Insens	NSC	A	[36,37]
TRPV2	NM_017207	Vrl1	ND	NSC	A	[36]
TRPV4	NM_023970	Trpv4	ND	NSC	A	[36]
TRPV6	NM_053686	Trpv6	ND	NSC	A	[36]

* gene was detected as present (P) or absent (A) in the presence of 100 nM dexamethasone.

** CNGA1 channels were not inhibited by 100 μM amiloride.

Insens, insensitive; ND, not determined; NSC, non-selective cation channels.

## Discussion

Several epithelial domains in the inner ear have been shown to absorb Na^+ ^either by itself or with K^+ ^from the lumen [4]. Highly-selective amiloride-sensitive Na^+ ^absorption has been shown in Reissner's membrane of the cochlea, while parasensory K^+ ^efflux was found in the outer sulcus epithelial cells of the cochlea and in the vestibular transitional cells. The present study is the first demonstration that the SCCD epithelial cells provide a highly-selective amiloride-sensitive Na^+ ^absorption pathway in the vestibular labyrinth that does not provide a route for parasensory K^+ ^efflux.

The [Na^+^] of vestibular endolymph is normally only about 10 mM, but rises rapidly during ischemic anoxia [9]. Local ischemic events occur in response to inflammation and other insults that release TNF-alpha (infection, autoimmune disorders, etc) [10]. Increased endolymphatic [Na^+^] can lead to cytosolic overload in hair cells [3] and vestibular dysfunction. Local release of TNF-alpha in the inner ear may have the additional exacerbating effect of reducing ENaC-mediated Na^+ ^absorption [11]. Dexamethasone may therefore have multiple mitigating effects by not only stimulating ENaC expression and activity, as shown previously [8,12], but also by releasing the inhibition from TNF-alpha [11,13]. These results point to a potential therapeutic utility of dexamethasone to correct elevated Na^+ ^levels in vestibular disorders associated with TNF-alpha-mediated inflammation.

Generally, ENaC is known as a highly Na^+^-selective channel with a Na^+ ^to K^+ ^permeability of > 80, but there are reports of ENaC being poorly- or non-selective to cations under some conditions and this can be due to either altered ENaC subunit stoichiometry or increased expression of unrelated nonselective cation channels (Table 2) [14].

*ENaC subunit stoichiometry and amiloride sensitivity*. The α-, β- and γ-ENaC subunits were all expressed in SCCD epithelial cells at the mRNA and protein levels, although the stoichiometric subunit association in the apical membrane is not known. Homomeric α-ENaC channels behave as nonselective cation channels [14,15]. On the other hand, heteromeric αβγ-ENaC is highly Na^+^-selective under some subunit combinations [14,16,17]. Heteromeric αβ-ENaC and αγ-ENaC are also highly Na^+^-selective [18,19] (Table 2).

The Na^+ ^current in SCCD is likely carried by heteromeric αβγ-ENaC or αγ-ENaC but not homomeric α-ENaC or αβ-ENaC, as judged by ion selectivity and amiloride sensitivity. Homomeric and heteromeric ENaC are pharmacologically defined through their inhibition by amiloride and its analogs [20]. The IC_50 _value for amiloride (Table 2) of homomeric α-ENaC channels is 0.17 μM [21], heteromeric αβγ-ENaC is 0.1 μM [16], αβ-ENaC is 4 μM, αγ-ENaC is 0.13 μM [19]. The IC_50 _value of I_sc _for amiloride was 0.47 μM for cultured primary SCCD epithelial cells [8]. The present study has found an inhibitory effect of amiloride at 100 μM (84%) that is similar to that found earlier (81%) [8]. The IC_50 _for SCCD is similar to the IC_50 _value of amiloride in Reissner's membrane (0.7 μM) [22] (Table 2).

### Cation selectivity of other channels

CNGA1 (cyclic nucleotide gated) channels were "Present" in our gene array of rat SCCD (GEO GSE6197), but "Absent" in mouse Reissner's membrane (GEO GSE6196), another inner ear Na^+^-absorptive epithelium. This is an amiloride-insensitive channel (not inhibited by 100 μM amiloride [23]) that has a low cation selectivity [24-27]. This channel therefore does not contribute to the Na^+ ^currents reported here.

HCN1 and HCN3 (hyperpolarization-activated cyclic nucleotide-gated) channels are "Present" in our gene array of rat SCCD. HCN channels conduct Na^+ ^and K^+ ^with permeability ratios of about 1:3 [28,29]. Despite this preference for K^+^, HCN channels carry an inward Na^+ ^current under physiological conditions [30]. There are no reports of amiloride sensitivity of HCN channels. ASIC1a (acid sensitive ion channels) are "Present" in our gene array of rat SCCD. IC_50 _value for amiloride of ASIC1a is 10 μM and P_Na_/P_K _is 6-13 [18,31,32](Table 2). The HCN1, HCN3 and ASIC1a channels therefore do not contribute to the Na^+ ^currents reported here.

### The culture of epithelial cells with permeable and impermeable support

A previous report on primary SCCD epithelial cells that were cultured on permeable supports demonstrated the block of I_sc _by amiloride (IC_50 _470 nM) and benzamil (57 nM) [8]. In the present study, we found that amiloride inhibited Na^+ ^current and did not inhibit K^+ ^currents (in both Ussing chamber recordings and whole cell recordings) when cells were cultured on permeable supports. These results are consistent with currents mediated by heteromeric ENaC. On the other hand, whole-cell currents passed K^+ ^as well as Na^+ ^and were insensitive to amiloride when cells were cultured on impermeable supports. These results are consistent with currents mediated by nonselective cation channels of an unidentified molecular origin. Homomeric alpha-ENaC is not a likely candidate since the currents were amiloride-insensitive.

These observations are consistent with a fundamental change in ion channel expression that was dependent on the culture substrate. Similar observations were made previously on other cells. Alveolar type II (ATII) cells also express highly-selective Na^+ ^channels (HSC) when cultured on permeable supports but nonselective cation channels when cultured on impermeable supports [33]. H441 lung adenocarcinoma cells also alter the Na^+ ^selectivity of their transport under the two conditions [34]. These results are a strong cautionary note for the culture of epithelial cells. Presumably there is a basolateral accumulation of a waste product that cannot diffuse adequately into the bath and triggers a change in channel expression.

## Conclusions

The results of this study support the conclusion that the amiloride-sensitive current in SCCD epithelial cells is highly selective for Na^+ ^and does not support the transport of K^+^. When this is combined with the results of previous studies on the amiloride-sensitive transepithelial current, it suggests that SCCD contributes to maintaining the low Na^+ ^concentration in normal endolymph but is not involved in K^+ ^homeostasis through this pathway.

## Methods

### Tissue preparation and electrophysiological recordings

#### Primary culture of SCCD epithelia

SCCD were dissected from neonatal Wistar rats (3-5 days), as described previously [8,12] and followed protocols in compliance with internationally recognized guidelines with approval by the Kansas State University Institutional Animal Care and Use Committee. Epithelial cells from SCCD, exclusive of common crus, were dispersed and seeded on Transwell permeable supports (6.5 mm diameter, Costar 3470, Corning, NY) or on glass cover slips (3 mm × 5 mm) and cultured with DMEM/F-12 medium (Invitrogen 12500-062, Carlsbad, CA) supplemented with 5% fetal bovine serum, penicillin (100 U/ml) and streptomycin (100 μg/ml) (Sigma-Aldrich #P-7539). The cells were used within 6 to 12 days after seeding and confluence of the primary cultures on permeable supports (4-6 days after seeding) was verified by measurement of transepithelial resistance. Cultures were treated with dexamethasone (100 nM) for 24 h before recordings of I_sc _or whole cell currents.

#### Ussing chamber recordings of the SCCD

I_sc _was measured as described previously [8] from confluent monolayers of SCCD in an Ussing chamber (AH 66-0001, Harvard Apparatus, Holliston, MA) modified to accept the Transwells, maintained at 37°C and connected to a voltage/current clamp amplifier (model VCC MC8, Physiologic Instruments, San Diego, CA) via Ag/AgCl electrodes and HEPES-buffered bath solution bridges with 3% agar. I_sc _was directly measured under voltage clamp (short circuit conditions) and both apical and basolateral side solutions were stirred by bubbling air.

#### Whole cell recordings of the SCCD

Cover slips or filter inserts with an SCCD epithelial monolayer were mounted in a chamber (150 μl) on an inverted microscope and perfused with bath solution at a rate of 150 μl/s. The patch pipettes were pulled in two steps from borosilicate glass capillaries (WPI, 1B150F-4) using a vertical puller (PP-83, Narishige) and had a resistance of 8-12 MΩ. After achieving a high-resistance seal (> 1.5 GΩ), the whole cell patch configuration was achieved by rupturing the cell membrane with suction; we were only successful about 1 time out of ~80-100 trials due to the thinness of the cells and the rigidity of the support. Electrodes were connected to an Axopatch 200A amplifier (Axon Instruments/Molecular Devices Corp., Sunnyvale, CA) at a low-pass filter bandwidth of 1 kHz (four-pole Bessel). Recordings were made at 36-37°C. Membrane currents were recorded with a Digidata 1322A (Axon Instruments) interface and pCLAMP 9 software (Axon Instruments) at a sampling rate of 10 kHz. The same software was used for the analyses. Stability of the patch was ascertained by monitoring the cell capacitance (C_cell_) and resistance (R_cell_) at the beginning and the end of recordings; C_cell _changed less than 1 pF during experiments. The initial C_cell _for cells on permeable supports was 20.9 ± 3.6 pF (n = 14 and on impermeable supports was 15.5 ± 2.0 pF (n = 13). The holding potential was 0 mV and the voltage command protocol consisted of 10 voltage steps with 20 mV spacing from -120 mV to +60 mV, 200 ms per step. Data are presented with command voltages corrected for liquid junction potentials (16.1 mV in Na^+^-rich bath and 20.4 mV in K^+^-rich bath, calculated with Axon Instruments implementation of the JPCalcW program by Peter H. Barry and additional ion mobility values, http://web.med.unsw.edu.au/phbsoft/mobility_listings.htm). Currents were normalized by cell capacitance as a measure of cell membrane surface area.

### Solutions and chemicals

All reagents were from Sigma-Aldrich, unless otherwise stated. Amiloride (no. A-7410) was predissolved in dimethylsulfoxide (DMSO), which was used at a final concentration of 0.1% DMSO. Amiloride was added to the apical side bath at a final concentration of 100 μM. Dexamethasone-cyclodextrin complex (#D-2915), was pre-dissolved in water.

#### Whole cell recordings of the SCCD

The impermeant anions gluconate^- ^and methanesulfonate^- ^were used to exclude any possible contributions from anions to the measured currents. The bath solutions were 1) Na^+^-rich-- (mM) 150 Na-gluconate, 0.25 MgSO4, 1 Ca-gluconate, 10 HEPES and 5 glucose, pH 7.4 adjusted with NaOH, 2) K^+^-rich-- (mM) 150 K-gluconate, 0.25 MgSO4, 1 Ca-gluconate, 10 HEPES and 5 glucose, pH 7.4 adjusted with KOH. The osmolarity of both bath solutions was 315-320 mOsm.

The pipette electrodes were filled with the following two solutions in each electrode; tip solutions dialyzed the cells and the back fill solutions made contact with the Ag/AgCl electrode. For use with the Na^+^-rich bath, ~10 mm of the pipette tip was filled with (mM): 135 NMDG-methanesulfonate, 15 Na-gluconate, 5 Mg-ATP, 1 EGTA and 10 HEPES, pH 7.4 and the backfilling solution was identical except that 15 Na-gluconate was replaced by 15 NaCl and the water-soluble dye fast green added for visualization. For use with the K^+^-rich bath, the pipette tip solution contained (mM): 135 NMDG-methanesulfonate, 15 K-gluconate, 5 Mg-ATP, 1 EGTA and 10 HEPES and the backfilling solution was identical except that 15 K-gluconate was replaced by 15 KCl.

#### Ussing chamber recordings of the SCCD

The composition of the basolateral side solution was (mM) 145 NaCl, 5 KCl, 1.2 MgCl2, 1.2 CaCl2, 10 HEPES and 5 Glucose, pH 7.4 adjusted with NaOH. The composition of the apical side solution was (mM) 150 NaCl, 0.25 MgCl2, 0.25 CaCl2, 10 HEPES, pH 7.4 adjusted with NaOH; in the substitution experiments, 150 mM NaCl was replaced by 150 mM KCl, pH 7.4 adjusted with KOH.

### Gene array analysis

Total RNA was extracted from control (n = 4) and dexamethasone-treated (n = 4) samples of SCCD primary cultures using RNeasy Micro Kit. The quality and quantity were determined as described previously [12]. Affymetrix microarrays were used to examine the expression of the genes investigated here by electrophysiology. Our methodology conforms to the MIAME (Minimum Information about a Microarray Experiment) guidelines and details are deposited with the data in [GEO: GSE6197] [6].

### Statistical analysis

Data were expressed as the mean ± S.E.M. (n = number of whole cell patches or inserts for I_sc_).

The significance of increases and decreases in current were determined by Student's paired or unpaired t-test. Differences were considered statistically significant at a level of P < 0.05 (Microsoft Excel).

## Competing interests

The authors declare that they have no competing interests.

## Authors' contributions

MY extensively analyzed data and contributed to writing the manuscript. TW carried out the patch clamp recordings. SRP carried out the Ussing chamber recordings; his new address is 112 Greene Hall, Department of Anatomy, Physiology and Pharmacology, College of Veterinary Medicine, Auburn University, Auburn, AL 36849. DGH assisted the study, including microdissection of tissues and Ussing chamber recordings. DCM conceived of the study, participated in its design and direction and contributed to writing the manuscript. All authors read and approved the final manuscript.
